# Supplementary figures and images for: Use of Endoscopic Scraper and Cell Block Technique as a Replacement for Conventional Brush for Diagnosing Malignant Biliary Strictures
Source: Cancers (Basel). 2022 Aug 27;14(17):4147. doi: 10.3390/cancers14174147 (PMC9454915; doi:10.3390/cancers14174147)

Supplementary Figure S1.

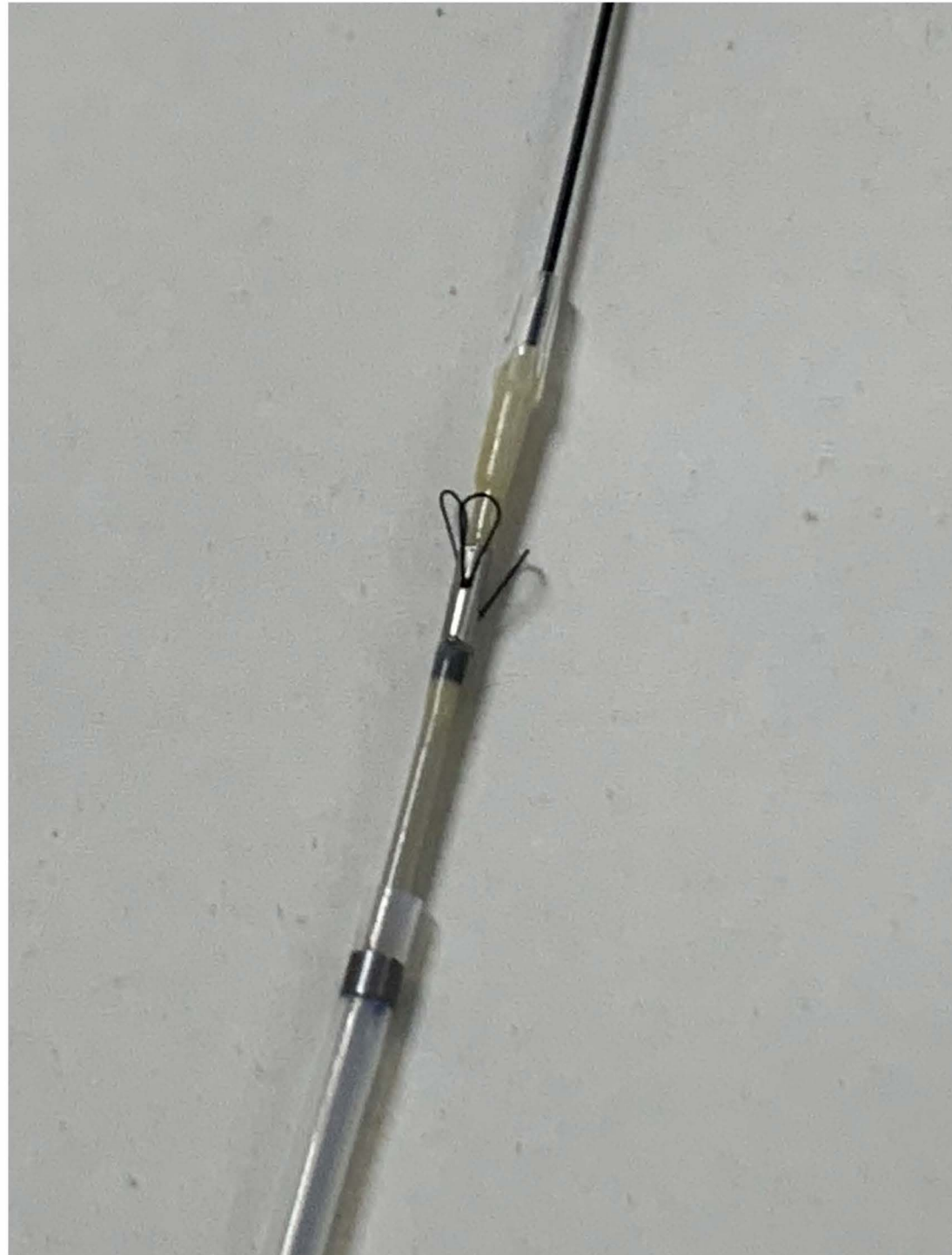

Supplement: Supplementary file 1 [file cancers-14-04147-s001.zip › Supplementary Figure S1.pdf]
